# Severe Panton–Valentine-Leukocidin-Positive *Staphylococcus aureus* Infections in Pediatric Age: A Case Report and a Literature Review

**DOI:** 10.3390/antibiotics13121192

**Published:** 2024-12-07

**Authors:** Valeria Garbo, Laura Venuti, Giovanni Boncori, Chiara Albano, Anna Condemi, Giuseppe Natoli, Valentina Frasca Polara, Sebastiano Billone, Laura Antonella Canduscio, Antonio Cascio, Claudia Colomba

**Affiliations:** 1 Department of Health Promotion, Mother and Child Care, Internal Medicine and Medical Specialties “G. D’Alessandro”, University of Palermo, 90127 Palermo, Italyclaudia.colomba@unipa.it (C.C.); 2 Department of Internal Medicine, National Relevance and High Specialization Hospital Trust ARNAS Civico, Di Cristina, Benfratelli, 90127 Palermo, Italy; 3 Division of Paediatric Infectious Disease, “G. Di Cristina” Hospital, ARNAS Civico Di Cristina Benfratelli, 90127 Palermo, Italy; 4 Infectious and Tropical Diseases Unit, AOU Policlinico “P. Giaccone”, 90127 Palermo, Italy

**Keywords:** Panton–Valentine leukocidin (PVL), *Staphylococcus aureus* (*S. aureus*), methicillin-resistant *Staphylococcus aureus* (MRSA), pediatric infections, necrotizing pneumonia, osteomyelitis, deep vein thrombosis (DVT), anti-toxin treatment

## Abstract

**Background:** Infections caused by S. aureus strains encoding Panton–Valentine leukocidin (PVL-SA) have become increasingly relevant in community settings and can cause severe conditions in pediatric populations. We present the pediatric case of an invasive disease caused by PVL-SA and provide a literature review of severe manifestations caused by these strains in children. **Methods**: A PubMed search (February 2024) found studies that included relevant clinical outcomes, diagnostics, and treatments, excluding cases of asymptomatic infection or in adult populations. A logistical multivariate analysis was used to find predictors of the need for intensive care. **Results**: A 10-year-old boy came to the attention of our Pediatric Infectious Diseases Unit with fever, chest pain, and tachypnea. A rapid worsening of his clinical conditions was observed, with the development of necrotizing pneumonia, osteomyelitis, deep vein thrombosis (DVT), and multiple abscesses. Blood cultures confirmed the presence of PVL-producing methicillin-resistant *S. aureus* (MRSA). The initial treatment included linezolid and ceftaroline and was later adjusted to clindamycin, daptomycin, and fosfomycin, with clinical improvement. **Discussion**: Our review collected 36 articles, including 156 pediatric cases of severe PVL-SA infection. Bacteremia was present in 49% of cases, lung infection in 47%, and osteomyelitis in 37%. The presence of pulmonary localization was predictive of the need for intensive care, O.R. 25.35 (7.46–86.09; *p* < 0.001). Anti-toxin molecules were used in about half the cases where information on treatment was reported. Our report highlights the capacity of PVL-SA to cause life-threatening complications in children, while also discussing the full range of its clinical spectrum and the most effective therapeutic approaches.

## 1. Background

*Staphylococcus aureus* (*S. aureus*) is a Gram-positive bacterium that frequently colonizes both human and animal skin and mucus membranes. It is the second bacterium, after *Escherichia coli* (*E. coli*), most commonly responsible for community-acquired bacteremia [1]. Further, *S. aureus* is the pathogen most often implicated in nosocomial infections and a significant cause of death in hospitalized patients [2]. The morbidity and mortality of *S. aureus* are mainly ascribable to the presence of genes enabling resistance to a variety of antibiotics, including the most effective and widely used anti-staphylococcal drugs. Until the mid-1990s, methicillin-resistant *S. aureus* (MRSA) infections were limited to hospitals, primarily affecting vulnerable individuals. However, over the past few decades, MRSA outbreaks have been reported in healthy individuals with no connection to healthcare settings. The presence of asymptomatic carriers facilitates the persistence and dissemination of staphylococci resistant to the most used antibiotics, especially in pediatric populations. Healthy carriers appear to be more at risk of developing invasive infections than non-carriers.

*S. aureus* can cause relatively benign skin infections such as folliculitis and furunculosis and life-threatening conditions such as deep-seated abscesses, osteomyelitis, pneumonia, endocarditis, and sepsis. Further, S. aureus can cause toxin-mediated diseases, such as toxic shock syndrome (TSS), staphylococcal scalded skin syndrome (SSSS), and food poisoning. Panton–Valentine leukocidin (PVL) is one of the main virulence factors of *S. aureus*, and it has become increasingly relevant over the past decade. It consists of two proteins (LukS-PV and LukF-PV), capable of assembling into an exotoxin on the cell cytoplasmic membrane of polymorphonuclear leukocytes, creating pores [3,4]. PVL-producing strains of S. aureus (PVL-SA) tend to cause more severe and harder-to-treat conditions and can spread rapidly in community settings where people spend time in close contact with one another, such as in schools and sports events. We present a severe case of invasive disease caused by PVL-SA and provide a scoping literature review of invasive infections caused by these bacteria in pediatric populations.

## 2. Results

### 2.1. Case Report

In June 2023, a previously healthy 10-year-old boy was admitted to the Emergency Room due to pain in his left knee that appeared after a football match. An X-ray was performed to rule out fractures, and, after the immobilization of the limb, the boy was discharged. Only two days later, the child was again admitted to the Emergency Room, due to the onset of fever, chest pain, and tachypnea, and subsequently transferred to the Pediatric Infectious Diseases Unit.

Upon admission, the patient was febrile (temperature 38.5 °C) and tachypneic, with a respiratory rate of 65 breaths per minute (bmp). A physical examination revealed a reduction in vesicular breath sounds at the base of the right lung, and a diffuse hyporesonance on percussion across all fields of the left lung. The abdomen was soft but tender to deep palpation. The left lower limb appeared painful, warm, and immobile.

Blood tests showed a white blood cell count (WBC) of 13.24 × 10^3^/µL, with a neutrophil count of 12.22 × 10^3^/µL, and a C-reactive protein (CRP) level of 52.77 mg/dL. A contrast-enhanced chest CT scan documented severe bilateral pneumonia.

On contrast-enhanced CT of the left femur, the presence of an effusion at the knee joint was documented, along with irregularities of the distal femoral metaphysis and liponecrosis of the distal third of the vastus medialis.

Color–Doppler ultrasound of the lower limbs showed a sub-occlusive thrombosis of the left posterior tibial, popliteal, superficial femoral, and common femoral veins, as well as thrombosis of the right posterior tibial vein. However, an extensive investigation into the patient’s coagulation profile revealed no genetically determined coagulopathy.

Antibiotic treatment with linezolid and ceftaroline was initiated, together with heparin.

A bronchoscopy with bronchoalveolar lavage (BAL) was performed and evidenced blood traces, aligned with the presence of necrotizing pneumonia. The BAL sample culture revealed the presence of MRSA, which was also isolated from blood cultures. The presence of genes encoding Panton–Valentine leukocidin (PVL) was evidenced through polymerase chain reaction (PCR).

On the sixth day of hospitalization, the patient underwent a magnetic resonance imaging (MRI) scan of the left lower limb, which documented the presence of osteomyelitis of the femoral diaphysis, arthritis of the knee, and extensive supra—and subfascial femoral abscess collections. A positron emission tomography (PET) scan also revealed osteomyelitis of the proximal right tibial epiphysis.

Immunological investigations were normal, including immunoglobulins, IgG subclasses, and lymphocyte subpopulations.

Due to the anticipated long treatment duration and in light of worsening anemia, linezolid and ceftaroline were discontinued, and clindamycin, daptomycin, and fosfomycin were initiated. The first was introduced because of its anti-toxin effect, and the latter two were added to ensure effective osteoarticular and pulmonary foci clearance. This change had a positive outcome with the progressive normalization of inflammation markers.

After a 4-week long antibiotic treatment, a follow-up chest CT was performed, and a reduction in the focal lung lesions was observed. However, pain and reduced mobility of the left lower limb persisted. Treatment was continued for an additional 4 weeks, and upon improvement in clinical conditions, the boy was discharged after the administration of a single dose of dalbavancin (18 mg/kg). A follow-up MRI, performed about two months after discharge, showed improvement in the femoral bone structure.

Notably, on the third day of the hospitalization of our patient, his seventeen-year-old brother, a professional wrestler, was admitted to our unit because of fever associated with widespread pustular lesions on his trunk and limbs. The culture of the drained material yielded PVL-SA. Upon screening of the household, PVL-SA was evidenced from nasopharyngeal swabs of the patient’s sister and cousin. Family members with MRSA-positive nasopharyngeal swabs were decolonized by applying nasal mupirocin for 5 days. The skin lesions of our patient’s older brother were treated with mupirocin ointment for 10 days.

### 2.2. Literature Review

**Figure 1:**
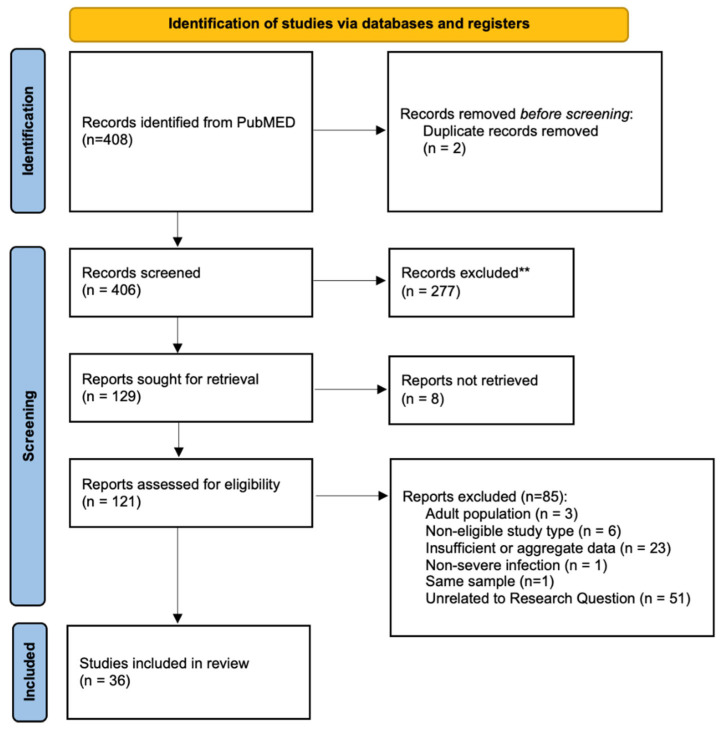
Flow chart describing the study selection process.

The search yielded 927 results without filters. After the application of filters, 408 results remained. The main reasons for exclusion were the non-eligibility of article types or population, the inability to extract data regarding pediatric populations or subgroups of children with PVL-SA infections, and the lack of relevance to the research question (studies that did not focus on PVL-SA infections). Thirty-six studies were therefore included in the final selection (Figure 1).

The main clinical–epidemiological data gathered from the selected studies are summarized in Table 1.

The collective number of cases gathered from the included studies was 156. In 56 cases, the gender was not specified. Of the remaining cases, 72 were boys (72%) and 28 were girls (28%). The mean age of the 137 patients for whom information was available was 5.9 years. Some authors reported aggregated data with group means, and this subsample was composed of 82 children. The mean age in this subgroup was 5.08 years (range 0.72 to 9.06 years), with a weighted standard deviation of 3.36 years. As for the group of individual patients, the mean age was 7.12 years (range 0 to 17 years), with a median of 8.3 years, and an interquartile range (IQR) of 0.875 to 12 years.

**Figure 2:**
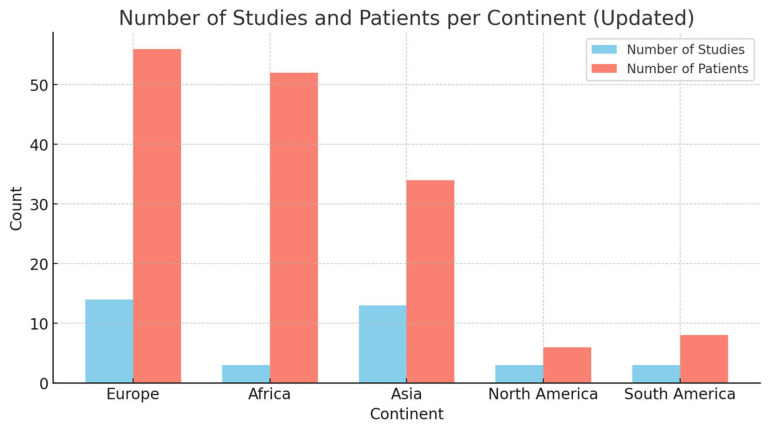
Bar chart describing the countries where patients were treated and/or where they likely contracted the infection. Fifty-six patients from 14 studies were observed in Europe, fifty-two from 3 studies in Africa, thirty-four from 13 studies in Asia, and six and 8 from North America and South America, respectively.

PVL was evidenced from MRSA strains in 61 cases (39%) and from MSSA strains in 95 cases (61%). Children most likely contracted the infection in the area where they were treated, except for four cases, where a history of recent travel was mentioned. In particular, 36% of cases were observed in Europe, 33% in Africa, 22% in Asia, and 4% and 5% in North and South America, respectively (Figure 2).

Immune compromission affected 21 children out of 89 cases where data were reported (23%). Comorbidities were present in 48 cases out of 108 where information was available (34%).

PVL-SA was identified from a variety of samples based on clinical presentation: nasal swab (*n* = 6), blood samples (*n* = 76), drained fluid or surgical samples (*n* = 86), broncho-alveolar lavage (*n* = 18), and sputum culture (*n* = 25). The affected organs and systems, with relative frequencies, are summarized in Table 2.

Information on complications such as deep vein thrombosis (DVT) and pulmonary embolism (PE) was extractable in 137 cases. Deep vein thrombosis (DVT) was reported in nine cases (6%) and pulmonary embolism (PE) in six cases (4%).

In 24% of cases (*n* = 37), the infection affected multiple systems. Lung foci and osteomyelitis were both present in 13 cases, and pulmonary and SST infections coexisted in 16 cases. In 11 cases, all three systems were affected.

Information on hospitalization length was infrequently reported (*n* = 68, 44% of cases). Among these cases, the mean length of hospital stay was 23 days, ranging from 1 to 88 days. Information on the infective status of the patient’s family members was reported in 18 cases (11%). Of these, in 11 cases (61%), some family members were infected as well, and in the remaining 7 cases (39%), the patient’s relatives were unaffected.

Thirty-four patients out of 63 cases where this information was reported (54%) needed intensive care. The presence of pulmonary localization was predictive of the need for intensive care, O.R. 25.35 (7.46—86.09; *p* < 0.001).

Treatment was detailed in 61 cases (39%). Of these cases, in 35 cases (57%), molecules with anti-toxin action were included during the treatment course.

Information on outcomes, including possible sequelae and relapses, was reported in most cases (*n* = 143, 92%). In 71% of cases (*n* = 102), a full recovery was described, or there was no mention of any sequelae or relapses. Fifteen children (10%) recovered with sequelae, and twenty-three were deceased (16%). In six cases (4%), patients recovered after one or more relapses.

The reported sequelae included the following manifestations: venous insufficiency and stenosis of the iliac vein, amputation (*n* = 2), chronic osteomyelitis (*n* = 2) and femoral necrosis, resolving hemiplegia, hemiparesis, unspecified functional impairment of the limbs, post-intensive care syndrome with reduced lung capacity and critical illness polyneuropathy, growth arrest (*n* = 2), pathological fracture, and suicidality.

## 3. Discussion

The case we describe is an example of the potential for acute and aggressive onset in PVL-SA infections. Our patient was a previously healthy child who, after being initially discharged with a leg cast, returned to the hospital within 48 h with deep vein thrombosis (DVT), a serious complication in PVL-associated infection. This highlights the capacity of PVL-SA to have a sudden onset with life-threatening complications. The boy’s condition progressed to bilateral necrotizing pneumonia, osteomyelitis of the left femur and right tibia, as well as a deep-seated abscess, all within a short period.

At the same time, this report serves as a reminder for the broad clinical spectrum associated with PVL-SA infections, ranging from mild presentations to life-threatening diseases. In contrast with our patient’s aggressive course, his older brother experienced only localized skin abscesses, and both his younger sister and cousin were asymptomatic carriers.

The reported case further highlights an important risk factor for PVL-SA infection: the involvement in skin-to-skin contact sports. In fact, the boy’s older brother, who was likely the primary carrier who transmitted the infection to the rest of the family, is a professional wrestler. Of the cases included in our review, a history of sports activities was reported four times (rugby [18,25], basketball, and soccer [38,39]), and a recent travel history was mentioned in five instances [19,20,32,36]. Of the cases in our review where gender was specified, 72% were boys. This could reflect the greater socialized tendency in boys to engage in contact sports or play that exposes them to skin-to-skin contact and a higher risk of superficial tissue injuries. Despite this, evidence shows that the female sex is linked with an increased mortality risk in patients with SAB [41].

PVL-SA infections have been associated with invasive disease, greater symptom severity, an increased risk of complications and ICU admissions, and longer hospitalizations [8]. However, publication bias should be considered when assessing the evidence of PVL-SA infection severity. The PVL-SA disease spectrum can range from SSTIs to musculoskeletal, pulmonary, cardiovascular, and CNS infections. Our patient had osteomyelitis and extensive abscess collections along with necrotizing pneumonia. Our review evidenced 13 similar cases where the infection affected the osteoarticular and respiratory systems. Pneumonia appears to be the most common manifestation of severe PVL-SA infections in pediatric age (47%), followed by severe SSTIs (38%), and osteoarticular localization (37%). When caused by non-toxin-producing S. aureus, pneumonia is generally less aggressive and most often associated with hospital-acquired infections [5,42]. Necrotizing pneumonia has been identified as the most severe of PVL-SA infection manifestations, often leading to ARDS and the need for mechanical ventilation [43]. In line with these findings, our analysis evidenced a strong association between pulmonary involvement and the need for intensive care (*p* < 0.001).

The prevalence of the PVL gene in S. aureus strains varies in different parts of the world and is higher in low- and middle-income countries.

The prevalence of PVL-SA colonization in the general population remains unclear. While several studies have investigated this in different countries, their findings are often subject to bias due to factors such as the lack of routine PVL testing and the focus on severe cases over minor skin and soft tissue infections [44]. PVL can be produced by either MSSA or MRSA strains. It has been reported that between 70% and 100% of isolated community-associated MRSA (CA-MRSA) strains carry the PVL genes, as opposed to only 9–46% of the MSSA strains [6,45,46]. Therefore, the prevalence of the PVL gene in S. aureus strains by country is influenced by that of MRSA as opposed to MSSA strains. Most invasive PVL-SA infections in the US are caused by CA-MRSA strains, while in Europe, the most commonly isolated PVL+ strains are those of MSSA [3,47,48,49,50]. Among the cases included in our review, PVL+ strains were more frequently MSSA (61%). In line with this finding, 36% of the cases in our review were from Europe, and only 4% and 5% were from North and South America, respectively.

Regarding the therapeutic approach, Gillet et al. underline the necessity to act against PVL-SA infections on multiple fronts: (1) source control, as in the removal of PVL through timely surgical drainage, (2) inhibiting its production, and (3) stopping its toxic effects post-production [43,50]. In the studies we reviewed, several different therapeutic regimens were employed, which was likely attributable to the lack of pediatric trials on PVL-SA infection treatment. The ARREST evidenced that the addition of rifampicin to standard antibiotic therapy does not significantly improve clinical outcomes in adult patients with SAB [51]. However, children were not included in this trial, and it did not specifically address PVL-SA infections. According to UK guidelines on managing PVL-SA infections, in pediatric cases with features suggestive of PVL-SA, clindamycin should be added to the first line empiric antibiotic treatment, which depends on the infection type and local epidemiology [52]. Linezolid should be added if MRSA is suspected or, importantly, upon evidence of PVL. In cases of bone and joint infections, recommendations include an initial treatment phase with clindamycin, linezolid, and rifampicin until the normalization of inflammatory markers (with linezolid treatment not longer than 4 weeks), and a continuation phase with clindamycin plus rifampicin, following a pediatric infectiologist’s guidance on duration and route [52]. Clindamycin, linezolid, and rifampicin can reduce PVL production [53]. These molecules also have the advantage of maintaining effectiveness even at suboptimal concentrations, while beta-lactams and vancomycin may aggravate PVL effects when below their MICs [53,54,55]. Despite these recommendations, since RCTs on the treatment of PVL-SA infection in children are lacking, clinicians should remain aware of the risk of overtreatment.

Our first-line treatment, motivated by the suspicion of PVL+ CA-MRSA, consisted of linezolid and ceftaroline. Upon the development of anemia, the confirmation of the presence of PVL, and confirmation of methicillin resistance, treatment was rationalized to clindamycin (for its anti-toxic effect and lung penetration), and fosfomycin plus daptomycin for their synergistic effect against S. aureus in bone infections [56]. Despite the lack of treatment data in the cases included in our review, available for 39% of cases, 57% of the described treatments included anti-toxin molecules.

As part of managing PVL-SA infection cases, investigations should be undertaken to identify a possible familiar and individual history of PVL-SA infections in the previous year [52]. To prevent reinfection/relapse, we treated our patient with a dose of long-acting antibiotics upon discharge and decolonized his infected family members. The role of decolonization treatment in the eradication of healthcare-associated pathogens such as *S. aureus* has been debated. Many defend the role of decolonization in households where recurrent CA-MRSA infections occur or where an immunocompromised individual resides. In the CLEAR trial, topical decolonization was more effective than hygiene education alone in reducing infection risks and readmissions for patients transitioning from hospital to home or other care settings. Commonly used topical agents include mupirocin, fusidic acid, and nasal mupirocin [57]. In patients with recurrent skin abscesses caused by PVL-SA, recurrence remains frequent even after decolonization efforts. Studies indicate that recurrence rates range from 30 to 60%, influenced by factors such as adherence to decolonization protocols, the colonization of family members or close contacts, and regional differences [58]. Surprisingly, in our review, information on familiar involvement was reported only in 18% of the cases we collected, which reflects a possible underestimation of the risk posed by family outbreaks. Familiar anamnesis is fundamental in that it could lead to the timely identification of PVL-SA in other possibly infected children and help prevent potentially severe relapses and the further spread of the infection.

## 4. Materials and Methods

We searched PubMED in February 2024 for articles describing pediatric cases of severe infections caused by PVL-SA in pediatric populations published since 2010. We used keywords such as “Panton-Valentine leucocidin”, “pvl”, “children”, “pediatric”, and relevant synonyms to broaden our results. Our main inclusion criteria were (1) a focus on pediatric populations, (2) evidence of PVL-SA strains as causative agents of the described conditions, (3) the severity and clinical significance of the infection, and (4) the clinical focus of the article, with information on manifestations and outcome. We excluded articles that (1) did not have a focus on children or where information on pediatric cases could not be extracted; (2) did not clearly report evidence of PVL-SA strains; (3) focused on asymptomatic carriers or contamination with PVL-SA; and (4) did not include information on isolated samples, methicillin resistance or sensitivity (MRSA or MSSA), clinical manifestations, and outcomes. To refine our search, we applied filters, selecting our population of interest and articles published in English. No automation tools were used.

Clinical and demographic characteristics were summarized through descriptive statistics, using mean, median, and interquartile range (IQR) for continuous variables, and absolute and relative frequencies for categorical variables. Logistical multivariate analysis was used to assess through odds ratios (OR) the correlation between the need for intensive care (dependent variable) and the variables relative to clinical manifestations (independent variables). These were selected according to the Hosmer–Lemeshow methodology. While this methodology provides an objective approach for variable selection, it has limitations, including its sensitivity to sample size and the reliance on arbitrary grouping, which could affect model calibration. After univariate analysis, only variables with *p* < 0.20 were included in the final model; then, through a backward elimination process dictated by the Hosmer–Lemeshow method, variables were excluded until a significance level of *p* < 0.05 was reached for each variable. The application of the test is a measure of how well the model fits the data without any variable selection by the researcher to be included in the multivariate model. A two-tailed *p*-value <0.05 was considered statistically significant. ‘STATA version 17.0’ was the database management and analysis software.

## 5. Conclusions

The case we described of PVL-SA infection in a previously healthy 10-year-old boy exemplifies how this condition can manifest abruptly, causing a severe, multiorgan disease in pediatric age. Given that the boy was likely infected by his teenage brother, a wrestler, and that a full-blown family outbreak occurred, this case sheds light on the importance of familiar anamnesis both for the identification of risk factors that can aid clinical suspicion and for the optimal management of the infection, which includes the decolonization of the household.

Necrotizing pneumonia, the main life-threatening manifestation of PVL-SA infection, is strongly linked with the need for ICU in our review, reinforcing pre-existing findings. The treatment of severe cases should include an anti-toxin molecule (such as clindamycin, linezolid, or rifampicin) and timely source control. If household decolonization proves ineffective, often due to resistance, and the patient has been hospitalized for a long time, making them fragile and at risk of relapses, long-acting antibiotics may be a reasonable option to provide additional protection.

## Figures and Tables

**Table 1:** Overview of the main clinical–epidemiological characteristics extracted from the included studies. * Age is expressed in years, and when cases are grouped together, the mean age is reported. Acronyms: ICU—Intensive Care Unit, MRSA—methicillin-resistant *Staphylococcus aureus*, MSSA—Methicillin-Sensitive *Staphylococcus aureus*, URTI—Upper Respiratory Tract Infection, RSV—Respiratory Syncytial Virus, CMV—Cytomegalovirus, PE—pulmonary embolism, SSTI—skin and soft tissue infections, DIC—Disseminated Intravascular Coagulation, RZHE—(likely a combination of antibiotics, but it is not standard; may require clarification), BAL—bronchoalveolar lavage, HL—hospitalization length, (d)—days, os—orally (in this context, medication administered orally), NR “not reported”, N/A “not applicable”.

Reference	Patients	Age *	Immune Compromission and Comorbidities	Samples Used for Diagnosis	Methicillin Sensitivity	Clinical Picture	Complications and ICU	Treatment	HL (d)	Outcome
Castellazzi et al. (2021) [5]	F (1)	0.5	Left bronchus malformation, history of apnea, URTI, bronchiolitis.	Blood, drainage material, BAL. *S. pneumoniae* and *Haemophilus influenzae* detected in the BAL culture	MRSA	Muscle abscesses, femoral osteomyelitis, lung nodules, pulmonary septic emboli, sepsis	PE, ICU	Cefotaxime ➔ Ceftaroline + Daptomycin + Clindamycin ➔ Ceftaroline ➔ Linezolid (os)	49	Full recovery
Albiński et al. (2018) [6]	M (1)	12	N/A	Blood	MSSA	Osteomyelitis (tibia), sepsis	Absent	Cefuroxime + Flucloxacillin + Gentamicin	39	Growth arrest
Albiński et al. (2018) [6]	M (1)	10	N/A	Blood and drainage material	MSSA	Osteomyelitis (humerus)	Absent	Amoxicillin-clavulanic + Clindamycin + Flucloxacillin ➔ Clindamycin (os)	16	Pathological fracture
Albiński et al. (2018) [6]	M (1)	12	N/A	Blood and drainage material	MSSA	Panosteomyelitis (femur, tibia)	ICU	Amoxicillin-clavulanic + Amikacin + Flucloxacillin + Vancomycin ➔ Levofloxacin + Rifampicin (os)	83	Suicidality
Albiński et al. (2018) [6]	M (1)	13	Acute Lymphoid Leukemia	Blood and drainage material	MSSA	Necrotizing pandiaphysitis (tibia), sepsis	Absent	Cefuroxime + Flucloxacillin	35	Growth arrest
Albiński et al. (2018) [6]	M (1)	10	N/A	Blood and drainage material	MSSA	Arthritis (hip)	Absent	Flucloxacillin + Gentamicin ➔ Clindamycin (os)	27	Full recovery
Albiński et al. (2018) [6]	M (1)	15	N/A	Blood and drainage material	MSSA	Arthritis (knee)	ICU	Amoxicillin-clavulanic + Clindamycin + Flucloxacillin + Imipenem + Vancomycin	74	Relapse
Albiński et al. (2018) [6]	M (1)	2	N/A	Drainage material	MRSA	Abscess (foot)	Absent	Amoxicillin-clavulanic + Clindamycin + Flucloxacillin	17	Full recovery
Albiński et al. (2018) [6]	F (1)	2	N/A	Drainage material	MSSA	Abscess (gluteal)	Absent	Amoxicillin-clavulanic + Clindamycin	1	Full recovery
Albiński et al. (2018) [6]	F (1)	12	N/A	Drainage material	MSSA	Abscess (finger)	Absent	Flucloxacillin	8	Full recovery
Moutaouakkil et al. (2022) [7]	M (12) F (5)	8,12	NR	Blood cultures, articular fluids, synovial tissues, and/or bone fragments	MRSA	Osteomyelitis (17), STTI (8)	Absent	NR	17.41	Full recovery
Hoppe et al. (2019) [8]	M (6) F (4)	5.73	URTI (RSV-B3, Influenza A) (4); Neonatal drug withdrawal; congenital CMV; heart transplantation.	Blood, pleural exudate, sputum, and bronchial lavage samples	MSSA (6) MRSA (4)	Necrotizing pneumonia (5), necrotizing fasciitis of the thorax (2), pyomyositis (2), axillary abscess (1), mastoiditis and cerebellitis (1), multiple and recurrent abscesses (1), preorbital cellulitis (1), purulent conjunctivitis (1)	DVT (2), ICU (4)	NR	22.6	FR (9), S (1); persisting post intensive care syndrome, including reduced lung capacity and critical illness polyneuropathy
Fujita et al. (2022) [9]	F (1)	0.3	N/A	Lumbar abscess drainage, nasal swab. Negative blood culture	MRSA	Extensive subcutaneous abscesses of the lumbar region	Absent	Cefotaxime ➔ Vancomycin ➔ Oral Sulfamethoxazole-trimethoprim	21	Full recovery
Karli et al. (2016) [10]	M (1)	12	N/A	Blood	MSSA	Multiple peripherally localized cavitary round lesions in both lungs (CT). Left psoas muscle abscess and left femoral trochanter osteomyelitis. Sepsis	PE, ICU	Vancomycin ➔ Linezolid + Clindamycin	30	Full recovery
Ahoyo et al. (2012) [11]	NR (19)	NR	Malnourishment after severe malaria, anemia, hospitalization	Pleural fluid (6), nasal swab (2), BAL (11)	MSSA	Pneumonia (19)	NR	NR	NR	Full recovery (2); Deceased (17)
Hardy et al. (2019) [12]	F (1)	4.6	N/A	Blood, drainage material	MSSA	Acute osteomyelitis, dermohypodermitis, septic arthritis, multifocal lesions, cardiac tamponade	DVT, ICU	Amoxicillin-clavulanic + Gentamicin ➔ Cefotaxime + Fosfomycin + Clindamycin	11	Deceased; cardiac tamponade
Hardy et al. (2019) [12]	M (1)	14.7	N/A	Blood, drainage material. *Staphylococcus haemolticus* in pericardial effusion	MSSA	Acute osteomyelitis, open skin wound	Absent	Cefotaxime + Gentamicin + Fosfomycin ➔ Clindamycin + Ciprofloxacin ➔ Ciprofloxacin (os) + Rifampicin (os)	14	Full recovery
Hardy et al. (2019) [12]	F (1)	11.2	N/A	Blood, drainage material	MSSA	Acute osteomyelitis, open skin wound	Absent	Amoxicillin-clavulanate + Gentamicin ➔ Clindamycin + Ciprofloxacin + Fosfomycin ➔ Ciprofloxacin (os) + Rifampicin (os)	18	Full recovery
Hardy et al. (2019) [12]	F (1)	13	Active chronic Hepatitis B	Blood, drainage material	MSSA	Acute osteomyelitis, dermohypodermitis, septic arthritis, multifocal lesions, necrotizing fasciitis, pyomyositis, necrotizing pneumonia	ICU	Cefotaxime + Gentamicin + Vancomycin ➔ Cloxacillin + Clindamycin + Rifampicin ➔ Clindamycin (os) + Rifampicin (os)	88	Functional impairment (ankle)
Hardy et al. (2019) [12]	M (1)	8.4	N/A	Blood, drainage material	MSSA	Acute osteomyelitis, septic arthritis, multifocal lesions.	Absent	Cefotaxime + Gentamicin ➔ Cefotaxime + Vancomycin ➔ Clindamycin (os) + Rifampicin (os)	20	Full recovery
Hardy et al. (2019) [12]	M (1)	6.5	N/A	Blood, drainage material, sputum culture	MSSA	Acute osteomyelitis, septic arthritis, multifocal lesions, necrotizing pneumonia	Absent	Cefotaxime + Gentamicin + Vancomycin ➔ Cloxacillin + Clindamycin ➔ Clindamycin (os) + Rifampicin (os)	42	Functional impairment, chronic osteomyelitis
Ogawa et al. (2022) [13]	F (1)	1	N/A	Surgical drainage	MRSA	Retropharyngeal abscess	ICU	Ampicillin/sulbactam + Vancomycin ➔ Clindamycin	22	Full recovery
Oshima et al. (2021) [14]	M (1)	0.08	N/A	Blood	MRSA	SSTI, necrotizing pneumonia, and cerebral infarction. Sepsis	DVT, ICU	Meropenem and Cefotaxime ➔ Vancomycin + Meropenem ➔ Linezolid	68	Right-sided hemiparesis
Vanbiervliet et al. (2022) [15]	M (1)	12	N/A	Blood	MSSA	Subperiosteal abscess, osteomyelitis (ankle), pyomyositis, septic cardiomyopathy. Sepsis	DVT, PE, ICU	Piperacillin-tazobactam + Vancomycin ➔ Flucloxacillin, Linezolid, Clindamycin, and Levofloxacin	42	Amputation; hospitalization was probably longer
Chen et al. (2014) [16]	F (1)	15	N/A	Pleural fluid and sputum	MRSA	Necrotizing pneumonia with cavitary lung lesions and bilateral pleural effusion. Sepsis	ICU	Linezolid + Fosfomycin + Teicoplanin	62	Full recovery
Schwartz et al. (2012) [17]	F (1)	0.67	N/A	Pleural fluid	MSSA	Necrotizing pneumonia, pulmonary hemorrhage	ICU	Cefotaxime ➔ Flucloxacillin + Vancomycin ➔ Linezolid + Rifampicin + Gentamicin ➔ Flucloxacillin + Rifampicin (after MSSA confirmed)	17	Deceased
Schwartz et al. (2012) [17]	M (1)	1.25	N/A	Blood, pleural fluid	MRSA	Necrotizing pneumonia, pulmonary hemorrhage, cerebral septic emboli and abscesses, erythroderma. Sepsis	ICU	Flucloxacillin, Cefotaxime, Ampicillin, and Azithromycin ➔ Hydrocortisone and IVIG ➔ Flucloxacillin + Vancomycin	28	Deceased
Schwartz et al. (2012) [17]	F (1)	0.67	N/A	Pleural fluid and tracheal aspirate	MRSA	Necrotizing pneumonia with multiple pneumatoceles	ICU	Clindamycin and Cefotaxime ➔ Vancomycin ➔ Clindamycin + Linezolid + Rifampicin	7	Full recovery
Schwartz et al. (2012) [17]	M (1)	0.42	N/A	Scalp lesion, endotracheal tube, and blood	MRSA	Necrotizing pneumonia, infarction of the right cerebral and cerebellar hemispheres, purulent subcutaneous scalp lesion with surrounding erythema. Sepsis	ICU	Cefotaxime, Flucloxacillin, and Vancomycin ➔ Linezolid + Lincomycin + Rifampicin ➔ Clindamycin	7	Resolving hemiplegia
Irenji et al. (2018) [18]	M (1)	13	N/A	Blood, abscesses material	MSSA	Extensive pelvic abscesses, bilateral pneumonia, pericardial effusion, and osteomyelitis. Sepsis	ICU	Flucloxacillin + Cefotaxime ➔ Linezolid + Clindamycin	41	Full recovery
Elledge et al. (2014) [19]	M (1)	10	N/A	Blood	MRSA	Osteomyelitis of the proximal tibia, history of boils on the buttocks and thighs.	Absent	Flucloxacillin + Rifampicin + Linezolid ➔ Flucloxacillin (os) + Rifampicin (os) ➔ Linezolid (os)	14	Full recovery
Cocchi et al. (2013) [20]	M (1)	0.25	N/A	Pleural drainage sample	MRSA	Necrotizing pneumonia, pyopneumothorax.	ICU	Ampicillin-sulbactam + Gentamicin (initial treatment; adjustments not mentioned)	NR	NR
Lehman et al. (2010) [21]	M (1)	6	N/A	Blood, abscess drainage material	MSSA	Necrotizing pneumonia and necrotizing fasciitis, septic osteomyelitis and arthritis, pulmonary consolidation. Sepsis	PE, ICU	Vancomycin + Cefotaxime ➔ Oxacillin + Gentamicin	42	Full recovery
Green et al. (2017) [22]	M (1)	13	Heterozygous factor V Leiden mutation	Blood	MSSA	Periorbital cellulitis	DVT	Ceftriaxone + Metronidazole ➔ Ceftriaxone + Clindamycin (os)	NR	Full recovery
Khattak et al. (2019) [23]	M (1)	0	Preterm infant (30th week)	Blood	MSSA	Cavitating pneumonia, shoulder abscess, cerebral abscess, osteomyelitis. Sepsis	ICU	Cefotaxime + Vancomycin ➔ Flucloxacillin + Linezolid	42	Full recovery
Gadelsayed et al. (2012) [24]	F (1)	11	N/A	Nasal swab, BAL.	MRSA	Cavitating pneumonia	Absent	Trimethoprim/sulfamethoxazole	NR	Full recovery
Fitzgerald et al. (2013) [25]	M (1)	14	N/A	Blood	MSSA	Discitis with an epidural abscess at L3–L4, necrotizing pneumonia. Sepsis	ICU	Flucloxacillin, Clindamycin, and Gentamicin ➔ Ceftriaxone, Clindamycin and Clarithromycin ➔ Linezolid added + IVIG ➔ Ceftriaxone and Clindamycin (oral)	NR	Full recovery
Karli et al. (2015) [26]	M (1)	12	N/A	Blood	MSSA	Necrotizing pneumonia, psoas abscess, cellulitis, and osteomyelitis. Sepsis	PE, ICU	Ceftriaxone + Vancomycin ➔ Vancomycin discontinued ➔ Linezolid ➔ Clindamycin (os)	30	Full recovery
Daskalaki et al. (2009) [27]	M (10) F (2)	2.3	N/A	Drainage material or skin samples (12)	MRSA (5) MSSA (7)	SSTI (12). Cellulitis, abscesses (9)	Absent	NR	NR	NR
Geng et al. (2010) [28]	NR (22)	0.72	Pneumonia with RSV (2), measles (2), CMV (1)	Sputum culture, pleural fluid and blood	MRSA (22)	Necrotizing pneumonia (22), complicated in 3 cases with empyema, pneumopyothorax, septicemia. Sepsis (1)	Absent	NR	NR	Ful recovery (21)
Kechrid et al. (2010) [29]	M (13) F (3)	6.18	N/A	Blood or bone tissues	MRSA (8) MSSA (8)	Osteomyelitis (10), bacteremia (6). Sepsis (1)	Absent	Oxacillin + Gentamicin (9); Teicoplanin + Gentamicin (3); Vancomycin + Gentamicin (1); Fosfomycin + Cefotaxime (1); Teicoplanin + Pristinamycin (2)	NR	Full recovery (14), sequelae (2); chronic osteomyelitis, necrosis of femur
Noguchi et al. (2021) [30]	M (1)	0.92	N/A	Sputum culture. Negative blood culture	MRSA	Necrotizing pneumonia, left-sided abscess and pyothorax, DIC	ICU	Cefotaxime ➔ Meropenem and Vancomycin ➔ Linezolid and Clindamycin ➔ Vancomycin and Clindamycin	31	Full recovery
Bybeck et al. (2020) [31]	NR (15)	9.06	N/A	Blood (15), and surgical drainage in cases with abscesses.	MRSA (2) MSSA (13)	Pulmonary localization (6); osteoarticular localization (5); SSTI (2); pericardial effusion (1); DIC (1); pneumothorax (2); respiratory insufficiency (4); one rhabdomyolysis and one local abscess. Sepsis (1).	DVT (1), ICU (4)	NR	NR	Full recovery (11); deceased (1); sequelae (3); venous insufficiency and stenosis of the iliac vein (1); above-knee amputation (1); chronic heart failure (1 case, unrelated to infection)
Mutale et al. (2014) [32]	M (1)	0.92	N/A	Blood, brain abscess sample. Negative nasal swab	MRSA	Parietal lobe abscess in the brain	ICU	Ceftriaxone + RZHE ➔ Vancomycin + Amikacin + Metronidazole + Cefotaxime + Rifampicin ➔ Rifampicin + Vancomycin + Linezolid	42	Full recovery
Bukhari et al. (2012) [33]	F (1)	0.75	N/A	Blood, drainage material	MSSA	Abscess formation and osteolytic lesions of the femur, left iliofemoral thrombosis	DVT	Ceftriaxone + Cloxacillin ➔ Clindamycin + Cloxacillin	56	Full recovery
Ambrozova et al. (2012) [34]	M (1)	0.83	*Citrobacter youngae* identified in stool culture	Blood, pleural fluid	MSSA	Pleuropneumonia and empyema, mediastinitis, SSTI. Sepsis	ICU	Cefotaxime ➔ Cefotaxime, Clindamycin, Gentamicin, Fluconazole, Corticosteroids ➔ Oxacillin + Clindamycin + Gentamicin	16	Deceased; progressive respiratory failure, pneumothorax, pneumoperitoneum, and circulatory failure, leading to death
Isobe et al. (2013) [35]	F (1)	17	N/A	Drainage material	MRSA	Osteomyelitis and multifocal pelvic (iliopsoas and piriformis) abscesses, adjacent to the sacroiliac joint.	Absent	Vancomycin + Pazufloxacin ➔ Post-discharge: Vancomycin + Minocycline (os) Second episode: Vancomycin + Fosfomycin ➔ Vancomycin (post-discharge) + Minocycline (os)	27	Relapse (3 episodes requiring hospitalization)
Obando et al. (2010) [36]	M (1)	12	Influenza A (H1N1) co-infection	Pleural fluid culture	MRSA	Necrotizing pneumonia and bilateral pleural empyema and pneumothorax	ICU	Ceftriaxone + Vancomycin + Clarithromycin ➔ Vancomycin + Clindamycin ➔ Linezolid + Clindamycin	28	Full recovery
Dunlop et al. (2011) [37]	M (1)	0.03	N/A	Cultures taken at the time of the first debridement.	MSSA	Necrotizing fasciitis	ICU	Cefotaxime + Gentamicin + Flucloxacillin ➔ Vancomycin + Clindamycin ➔ High-dose Flucloxacillin + Clindamycin + Gentamicin	35	Full recovery
Whitaker et al. (2023) [38]	M (1)	15	N/A	Blood, drainage materials, nasal swab	MRSA	Abscess in left psoas, subdural empyema in the sacral region extending to the lumbar spine	PE	Vancomycin ➔ Daptomycin ➔ Sulfamethoxazole/trimethoprim (os)	45	Full recovery
Higuchi et al. (2010) [39]	M (1)	7	N/A	Drainage material	MRSA	Abdominal cellulitis	Absent	Cefdinir (os)	7	Relapse
Kefala-Agoropoulou et al. (2010) [40]	F (1)	10	N/A	Blood, drainage material	MRSA	Pneumonia, signs of encephalopathy, severe osteomyelitis on the whole right femur and pyomyositis and thrombosis of the right femoral and right external iliac vein. Sepsis	DVT, ICU	Cloxacillin ➔ Vancomycin + Clindamycin + Gentamicin ➔ Teicoplanin + Clindamycin (os)	56	Full recovery

**Table 2:** Organs and systems affected by PVL-SA infection, with absolute and relative frequencies. Children were often affected by more than one manifestation.

Bloodstream (bacteremia)	76 (49%)
Respiratory system	73 (47%)
Skin and soft tissues	59 (38%)
Osteo-articular system	58 (37%)
Central nervous system	9 (6%)
Cardiovascular system	6 (4%)

## Data Availability

This study is based on secondary data from existing literature, and all data necessary to understand and interpret the findings are included within the manuscript.
